# Treatment of Asiatic Cholera

**Published:** 1895-10

**Authors:** Elmer Lee

**Affiliations:** Chicago.


ARTICLE II.

### TREATMENT OF ASIATIC CHOLERA.

BY ELMER LEE, A. M., M. D., PH. B., CHICAGO.

Read in the Section on Practice of Medicine. at the Forty-sixth Annual Meeting of the American Medical Association, at Baltimore, Md., May 7-10, 1895.

Spasmodic cholera—called also malignant, epidemic, Asiatic, Indian, blue, and pestilentical cholera—is generally epidemic, though not contagious. The first symptoms are generally experienced during the night, sometimes beginning with a light general uneasiness and moderate diarrhea; at other times the symptoms come on violently and follow each other rapidly. In fatal cases death usually occurs at some period between six and twenty-four hours; in a few fatal cases the patient lingers two or three days. The ordinary course of symptoms are more or less diarrhea; the discharges at first feculent, but soon presenting the appearance of rice-water or gruel; there are flying pains, or sense of coldness in the abdomen, as if purgative medicine were about to operate; the countenance is pale; there is nausea, vomiting, prostration of muscular power, and nervous agitation; cramps in the legs, arms, loins and abdominal muscles, more or less severe; small, weak pulse, intense thirst, and urgent desire for cold water; in most cases cold, clammy skin; all these symptoms may appear successively or almost simultaneously. In some cases the premonitory symptoms exist for eight or ten days; and sometimes the patient is prostrated at once. When the disease comes on suddenly the cramps usually begin in the fingers and toes, rapidly extending to the trunk; the eyes are sunken and surrounded by a dark circle; there is vomiting and purging of white matters mixed with flocculi; the features are sharp and contracted; the expression of the countenance wild and confused. The face, extremities, and often the whole surface of the body manifest a varying intensity of a leaden, bluish or purplish hue; the extremities shrunken, the nails blue, the pulse thready or wholly imperceptible at the wrist, arm, axilla, temple or neck; there is great restlessness, incessant jactitation, severe pain in the epigastrium, loud moaning or groaning, difficult and oppressed breathing; difficult inspiration, with short and convulsive expiration; voice hoarse, whispering, or nearly suppressed and plaintive; the tongue is white, cold and flabby, and the external temperature often sinks below 80 degrees; convulsions recur at short intervals, or a constant tremor exists. The secretions of bile, saliva, tears and urine are entirely suppressed, and a cadaverous odor exhales from the body. The patient retains his faculties to the last.

Some of the symptoms may be disproportionately severe, or may be entirely absent. Those usually regarded as pathognomonic are: watery dejections, blue appearance of the countenance or surface, thirst, coldness of the tongue, and pulselessness at the wrist.

The foregoing description of the symptoms of cholera is indicative of the nature of the disease calling for human aid. The time in which to treat the patient sick with cholera is exceedingly limited What is to be done must be executed with rapidity. There is not a moment to lose between the time when the patient is first seen and the accomplishment of severely practical efforts. Many wise theories may be promulgated, but there are few practical measures that will avail against Asiatic cholera. The experiences during the cholera epidemic of 1892 in Europe, both in Russia and Germany, produced in me a profound conviction that, for the most part, remedial agencies that have been used are of questionable utility. Nearly every prominent remedy proposed and tried has been found to end in greater or less disappointment.

Years ago, great reliance was placed upon the far-famed "mild chlorid of mercury." Twenty and ten years ago this remedy was given in large doses. Three years ago, during the latest epidemic, small doses prevailed. Next to this, the synthetic drug salol, the product of the laboratory of the Imperial Institute of Experimental Medicine in St. Petersburg, was the most widely used and the most favorably received. Professor Nenski, the originator of salol, personally informed me that the value of the drug could not be seriously recommended as of much importance, but that it perhaps answered the requirements as far as any drug could answer, in the hands of his colleagues. Widely circulated and various reports, enthusiastically commending and moderately commending this remedy were received by the Professor in St. Petersburg, but he himself was silent as to its efficacy. The far-famed and seemingly unmatched drug, quinin, has been used, and has been held as a dazzling gem before the eyes of the profession by some of our best men, who believe that cholera is analogous to malarial disorders, and consequently the medicine which occupies the position of keystone in the arch, for malarial treatment, is a remedy suitable to contend with the rapid and desperate symptoms of Asiatic cholera. Quinin has a stout advocate in our own country, in the person of a well-known professor in one of the Ohio medical colleges. It was not used, to my knowledge, in the treatment of cholera during the last epidemic in Europe.

A remedy was brought to Hamburg during the latter part of the epidemic of 1892 by the representative of an English syndicate, who posed as a chemist, not a physician. His remedy was a preparation of iodin, to be administered through the mouth. He called the medicine a periodate, and made some experiments upon patients in one of the cholera hospitals in Hamburg. His remedy, however, was not favorably entertained by the medical authorities in charge of the cholera patients, and whatever claims were reported came through the interest of a friendly correspondent of one of the Hamburg weekly secular papers. To show how misleading some of our supposedly authentic information often is, it is only necessary for me to refer to the report given in the "Year Book of Medical Progress," published in Philadelphia. Of all the progress made, of all the combined investigations during the entire epidemic of cholera throughout Europe in 1892, and there was an immense amount of original investigation and great effort made to discover a remedy, the curious spectacle in the Year Book, which alone refers to the remedies brought by an agent of a syndicate from London to Hamburg, at the closing of the epidemic of cholera, shows that there are some things in our profoundest medical publications that are to be taken *cum grano salis.* Uretin was extensively used hypodermically for its alleged influence upon the secretions of the kidneys, upon the ground that the kidneys were to be aided by irritating them to greater functional activity to eliminate morbid elements through the urine. The result of many investigations recorded in Russian practice show that this drug is not to be commended. Digitalis was used, supposedly to benefit a weak heart. This remedy; if at all useful, could be little more than palliative. The use of acidulated water was extensively employed in different hospitals in Europe as a drink, but not prescribed as a remedy. The water was acidulated with HCl and $H_2SO_4$. Subcutaneous injections of salt water were made. The proportion of salt was one-half of 1 per cent., and the amount of salt water injected subcutaneously was sometimes as much as a quart at a single injection. In one instance, during an illness of several days, as much as thirteen quarts was subcutaneously injected into the cellular tissue, principally that of the abdominal wall. This process of subcutaneous injection was known as hypodermaclysis. The purpose of the hypodermaclysis was to maintain the volume of the blood. The diminished volume of the blood is directly the result of the waste of its liquid portion or serum into the alimentary canal. In this serous discharge, flakes of intestinal mucous gave the name of "rice-water discharges" to the bowel evacuations, the particles having a resemblance to grains of rice. The general inflammatory state of the intestinal mucous membrane, throughout its entirety, drains the blood of its liquid portion rapidly, and collapse due to stagnation of circulation quickly ensues.

The remedies mentioned are only a portion of those tried, but there is no living advocate who to-day can point with unerring certainty to one single organic or inorganic substance, howsoever administered, that can be safely depended upon in the treatment of Asiatic cholera. Both botany and mineralogy have been searched in vain for a cure for this disease.

The cause of this disease is perhaps accurately stated to be due to invasion of the blood and, secondly, of all the tissues of the living organism, by toxins or ptomaines, which originated in the upper portion of the small intestine at the early stages of cholera. These products of organic activity, whether of animal or vegetable organisms it is here unnecessary to debate; but these noxious products enter the circulation through the villi of the intestine and rapidly and desperately poison the blood. It is clearly proved that the disease is the result of general blood poisoning from an intestinal origin. Whatever the chemic nature of the poison may ultimately be found to be, may be safely left to the bacteriologic laboratory. The practical and intensely important part that remains for physicians seeking to cure patients in times of this disease is to realize how much, as well as how little, it is within human power to do. The human organism is prostrated by a fierce and deadly poison. This poison is in the blood and in the cells of the tissues, and its work of destruction is quickly and effectually accomplished. Reflectively, to say nothing of experimental research, it would seem to me that the rational and only course that could be advocated with scientific assurance of relief is to, as far as possible, literally cause to be removed these products which are death-dealing to the body in which they happen to be found. Now, in this same reflective mood, think for a moment and try with me to determine whether it is possible in such conditions as produce the symptoms of Asiatic cholera, it is safer to introduce other poisonous products to neutralize the noxious elements in the blood and cells, or whether it is a better process to, without the introduction of additional foreign substances, remove what we already find in the blood. To make this proposition clearer, it could be stated in another way, namely, the body is already bearing a crushing burden; shall we add other foreign substances as an additional burden to the load already carried? The principle seems to me to be at fault. The principle is the principle of allopathy, but in the light of facts is it a safe principle to follow? It is reasonably scientific to produce in the laboratory, definite results in vessels of glass by the use of fixed reagents; in the organic laboratory of the living body, no such definite results can be demonstrated. The vital principle is an entity which enters into the formula and may be represented by the unknown quantity *x* in algebraic equations. Great and laudable efforts have been made to prevent as well as to cure this disease by inoculation.

Ferran, of Valencia, Spain, thrilled the world ten years ago with his proposition of a universal cure for this disease. His glory was then at its zenith. His fame has long since faded. So obnoxious became his proposition to the government of Spain that laws were adopted to suppress Ferran's cholera inoculations.

A worthy colleague and laborious investigator, Professor Haffkin, of Pasteur Laboratory fame, proposed a modified inoculation for the prevention and cure of cholera in 1892. A reporter of the New York *Herald* was inoculated at the Pasteur Institute, and with credentials sent to expose himself to Asiatic cholera at Hamburg in September, 1892. The same reporter had been similarly inoculated by Ferran in 1886 and had the courage to make further exploits in behalf of his newspaper, at Hamburg. A very widespread opinion prevails in America that the exploit of the New York *Herald* reporter during the ten days' stay as a nurse in the Hamburg hospital, constitutes a proof of the validity of Haffkin's claim, but the scientific world of Europe knows differently. *En passant*, it may be interesting to state at this place that further experiments have been made by Professor Haffkin in India with the cholera inoculations and, unfortunately for the proposition, reports have recently come to me from reliable medical sources, that a greater percentage are attacked with cholera who have been previously inoculated than of those who have not been inoculated. This subject of prevention, however, is to be discussed by me in a paper to be read before the Section on State Medicine.

The result of prolonged reflection, covering many years, and the observations resulting from personal experience in the cholera epidemic in Europe of 1892, is the conviction that there is provided in the laboratory of the universe a remedy which surpasses the results of human ingenuity as much as does the sun surpass in brilliancy the light of the artificial lamp. The all-pervading and all-wide remedy, the greatest product of omniscient nature's laboratory, which alone can cope with this pestilential disease of the human race, is nothing more and nothing less than the unmatched, unmatchable $H_2O$. Pure water is absolutely the only trustworthy cure for cholera, and if it came at a great price it would probably be more greatly valued. The human organism is so constituted that if it is assisted by $H_2O$, every morbid element may be eliminated out of its domain. The acutely poisoned body quickly recovers its equilibrium and its harmony of action as soon as the processes of elimination can remove the invading poison. In the construction of the mucous lining of all the accessible cavities and channels it is prepared by an undiscernible law to successfully resist the entrance of every form of organism. The products of organic action alone are able to pass into the blood. If sufficient quantities of pure water, of a suitable temperature, are introduced into the body through the natural channels, it is actually possible to wash morbid products as well as organic forms of life, out of the human body. The mouth gives entrance to the caustic germs in Asiatic cholera. That is quite conclusively established. The locality of the development and formation of the toxin in the earlier stages is determined to be in the upper end of the small intestine; and from experience, as well as from the powers of reflective analogy, there is no doubt that the system can be saved from death if the morbid entity, the germ, is literally deluged away from the alimentary canal by the copious use of a remedy that can not be of the slightest danger to the victim. The amount of water to be used varies in different cases. It is impossible to use too much; it is possible to use too little. From the earliest moment that the patient is seen, the propositions should be, first, wash the whole alimentary canal with pure water; wash the lower portion by introducing irrigations of warm soapsuds or merely warm water into the colon sufficiently frequently and sufficient in quantity to cleanse that portion of the bowel effectually; The frequency of washing that portion of the bowel which is accessible from the rectum should be one, or two, or three, or four times a day, according to circumstances. At the same time from one to ten quarts of warm pure water mildly medicated with peroxide of hydrogen or hydrozone should be administered at regular intervals, during the day, as the prescribed remedy by the mouth. If the patient vomits, very well. Immediately re-introduce the quantity of water that was vomited. No harm can be done in any case, and if it is possible to save life it is possible to save it through this method. It is the quickest and the surest method of exciting the activity of the kidneys, and is the safest. It is the rational and effective measure for maintaining the volume of the blood. It is the scientific process by which to establish cutaneous circulation in the capillaries.

The use of simple and useful hygienic measures are the same as in other prostrating diseases. Patients should be fed with regularity at not too frequent intervals, giving the proper time, between administrations of simple food, for its digestion. The use of appliances for maintaining the heat of the body are not to be neglected.

The precise details of the method of treatment indicated at this time will be forthcoming in a subsequent paper.—*Journal of the American Medical Association*, June 22, 1895.

---

ARTICLE III.

